# Gapless vortex bound states in superconducting topological semimetals

**DOI:** 10.1093/nsr/nwac121

**Published:** 2022-06-24

**Authors:** Yi Zhang, Shengshan Qin, Kun Jiang, Jiangping Hu

**Affiliations:** Department of Physics, Shanghai University, Shanghai 200444, China; Kavli Institute of Theoretical Sciences, University of Chinese Academy of Sciences, Beijing 100190, China; Kavli Institute of Theoretical Sciences, University of Chinese Academy of Sciences, Beijing 100190, China; Beijing National Laboratory for Condensed Matter Physics and Institute of Physics, Chinese Academy of Sciences, Beijing 100190, China; Kavli Institute of Theoretical Sciences, University of Chinese Academy of Sciences, Beijing 100190, China; Beijing National Laboratory for Condensed Matter Physics and Institute of Physics, Chinese Academy of Sciences, Beijing 100190, China; Collaborative Innovation Center of Quantum Matter, Beijing 100190, China

**Keywords:** superconductivity, superconducting vortices, topological semimetals, Andreev reflection

## Abstract

We find that the vortex bound states in superconducting topological semimetals are gapless owing to topological massless excitations in their normal states. We demonstrate this universal result in a variety of semimetals, including Dirac and Weyl semimetals, three-fold degenerate spin-1 fermions, spin-3/2 Rarita-Schwinger-Weyl fermion semimetals and other exotic fermion semimetals. The formation of these gapless bound states is closely related to their Andreev specular reflection and propagating Andreev modes in π-phase superconductor-normal metal-superconductor junctions. We further demonstrate that these gapless states are topologically protected and can be derived from a topological pumping process.

## Teaser

The authors demonstrate the existence of gapless vortex bound states in various superconducting topological semimetals, which is a universal property due to topological massless excitations in their normal states.

## INTRODUCTION

The topological semimetals (TSMs), such as Weyl and Dirac semimetals where their low-energy physics is governed by topological gapless excitations protected by topology and symmetry [1,2], have attracted much interest. Recently, by studying all space-group symmetries, new additional types of TSMs have also been classified [2–5], including spin-1 excitation [6], the spin-3/2 Rarita-Schwinger-Weyl (RSW) fermion [6–9], etc. TSMs host some fantastic properties, such as Fermi arcs and chiral anomaly related transports, etc. [1,10–22]. However, these exotic properties can be very different in different types of TSMs. Until now, there is no single universal property associated with all TSMs.

A vortex is a topological object in real space and is a central ingredient for a type-II superconductor (SC) under an external magnetic field [23,24]. For a conventional Fermi liquid with fully gapped pairing, the SC pairing function forms a quantum well inside a vortex core to generate numerous gapped bound states, named Caroli–de Gennes–Matricon states [25]. In this paper, we show that the vortex bound states (VBSs) in superconducting TSMs differ fundamentally from conventional Fermi liquids. These bound states are gapless in the vortices, which is a universal property for TSMs beyond Dirac semimetals (DSMs) and Weyl semimetals (WSMs) [26–30]. These states are topologically protected due to the topological gapless excitations in their normal states. The formation of these states is also closely related to the Andreev specular reflection and the propagating Andreev modes in π-phase SC-normal metal-SC (SNS) junctions.

**Figure 1.**
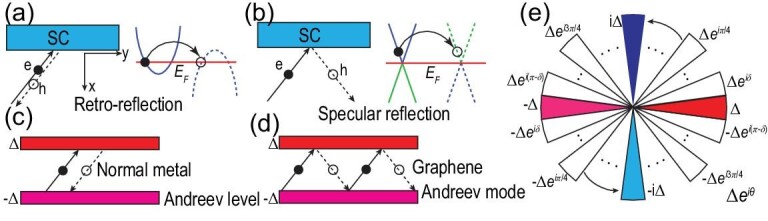
Schematic illustration of the Andreev reflection and its relation to vortex. (a) Retro-reflection in the conventional-metal-to-SC interface, where one electron is injected with one hole with opposite velocity reflected. (b) Specular reflection in the graphene-to-SC interface, where the reflected hole retains its velocity along the reflection plane. (c) Andreev level or Andreev bound state in an SNS junction owing to the multiple retro-reflections at each interface. (d) Propagating Andreev mode in a graphene SNS junction owing to specular reflections. (e) By slicing the pairing function Δ*e*^*i*θ^, the vortex can be mapped to infinite slices of π junctions by taking δ → 0. The π junctions along the *x* and *y* axes are highlighted.

To illustrate the main idea, we consider superconducting graphene as an example [31,32]. In a conventional Fermi liquid, if we inject one electron into the SC, one hole comes out, named the Andreev retro-reflection [33], as shown in Fig. 1(a). Since the reflected hole is from the conduction hole band, its velocity is opposite to the electron velocity from the conduction electron band. However, in graphene, due to the topological gapless nature, the reflected hole can sit in the valence hole band when the Fermi energy *E_F_* is around the Dirac point. Since the valence band has a different Fermi velocity compared with the conduction band, the hole can retain its velocity along the reflection plane, named the specular Andreev reflection [34], as shown in Fig. 1(b). The specular reflection leads to an unconventional behavior in the π-phase SNS junction in graphene [35,36]. Conventionally, the localized Andreev bound states can be found in the normal metal π junctions, as illustrated in Fig. 1(c) [37]. On the contrary, owing to specular Andreev reflection, the graphene π junctions show propagating Andreev modes, as shown in Fig. 1(d). The physics has been generalized to the superconducting topological insulator surface states in the seminal Fu-Kane proposal [38], in which a tri-junction is constructed for Majorana zero mode manipulation. This tri-junction can be viewed as a discrete analog of the superconducting topological insulator surface state vortex.

The above physics can be extended to a superconducting vortex. In a continuum model for graphene, a superconducting vortex hosts zero-energy bound states [39,40], which are closely related to zero modes bound to topological defects in the context of high-energy physics [41,42]. For a vortex, the order parameter can be written as Δ(*r*)*e*^*i*θ^, where θ is the phase angle centered at the vortex core. The gap value changes its sign by a 180° rotation. Therefore, we can slice the vortex into infinite numbers of π junctions, as illustrated in Fig. 1(e). The vortex is just the superposition of all the π junctions, which is similar to the quasiclassical VBSs approach [43]. Hence, we can conjecture that if the propagating Andreev modes exist in the π junction, there should be exotic bound states in the graphene vortex. Furthermore, it is also reasonable to argue that the specular reflection should be a common feature for all other topological semimetals.

## VORTEX BOUND STATES IN TOPOLOGICAL SEMIMETALS

**Figure 2.**
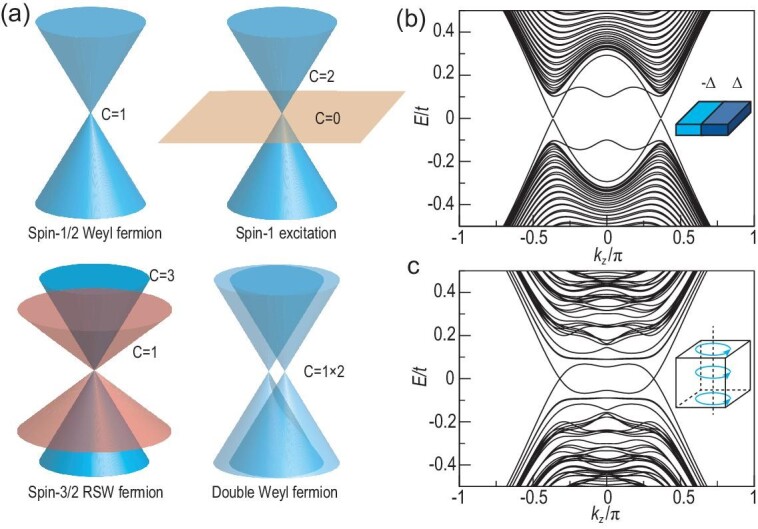
(a) Energy dispersions for multiple types of fermionic excitations, including the spin-1/2 Weyl fermion, spin-1 excitation, spin-3/2 RSW fermion and double-Weyl fermion. The Chern number C for each band is also labeled for each fermion. (b) The π-SNS junction spectrum along the *k_z_* direction for the DSM at *k_y_* = 0. There are gapless dispersions around the Dirac points. (c) VBS spectrum for the DSM. A similar gapless energy dispersion is obtained.

To confirm this conjecture, we consider various new types of fermionic semimetals in three-dimensional solids. Besides the spin-1/2 Weyl fermion, the spin-1, spin-3/2 massless fermionic excitations and the double-Weyl fermion are shown to exist owing to space-group symmetry [2,18], as illustrated in Fig. 2(a). Their low-energy Hamiltonian [2,18] can be written as


(1) \begin{eqnarray*} H=\delta \mathbf {k} \cdot \mathbf {S}, \end{eqnarray*}

where $\delta \mathbf {k}=\mathbf {k}-\mathbf {k}_0$ is the momentum deviation from the crossing point $\mathbf {k}_0$ and $\mathbf {S}$ stands for the pseudospin operator matrices in each spin representation. From Fig. 2(a), we can see that if *E_F_* is close to the degenerate points, the specular reflection always exists. To show this, we first take the DSM as an example.

The Hamiltonian for a DSM can be written as


(2) \begin{eqnarray*} &&\!\!\!\! H_{D}(\mathbf {k})=(m-t\cos k_x-t\cos k_y-t_3\cos k_z)\sigma _z \\ &&\quad+\,t\sin k_x\sigma _x s_z +t\sin k_z(\cos k_x-\cos k_y)\sigma _x s_x \\ &&\quad-\,t\sin k_y \sigma _y +2t\sin k_z \sin k_x \sin k_y \sigma _x s_y, \end{eqnarray*}

where the Pauli matrices σ_*i*_ and *s_i_* with *i* = *x, y, z* act in the orbital and spin spaces, respectively [26]. We set the parameters as {*t, t*_3_, *m*} = {1, 0.5, 2.2}. Hamiltonian *H_D_* hosts two Dirac points at $(0,0,\pm k_z^c)$ with $k_z^c=\arccos (0.4)$. With an *s*-wave SC, the Hamiltonian can be written as


(3) \begin{eqnarray*} H_{\rm SC}= \left({\begin{array}{cc}H_D(\mathbf {k})-\mu &\quad \mathbf {\hat{\Delta }(\mathbf {r})} \\ \mathbf {\hat{\Delta }^\dagger (\mathbf {r})} &\quad -H_D^T(-\mathbf {k})+\mu \end{array}}\right), \end{eqnarray*}

in the basis $\Psi _\mathbf {k}=(c_{\mathbf {k}\uparrow },c_{\mathbf {k}\downarrow }, c_{-\mathbf {k}\uparrow }^{\dagger },c_{-\mathbf {k}\downarrow }^{\dagger })^T$, where μ is the chemical potential and $\mathbf {\hat{\Delta }(\mathbf {r})}$ is the *s*-wave pairing function with $\hat{\mathbf {\Delta }}(\mathbf {r})=\mathbf {\Delta (\mathbf {r})}is_y$. Following the conventional Bogoliubov-de Gennes approaches to vortex bound states [25,44], we have neglected the effect of the magnetic field, which is negligible and irrelevant to vortex bound states.

We consider a π-junction structure with length *L* for the DSM along the *x* direction. The pairing function is defined as


(4) \begin{eqnarray*} \mathbf {\Delta (\mathbf {r})}= \left\lbrace \begin{array}{@{}l@{\quad }l@{}}\Delta _0, & x\le L/2, \\ -\Delta _0, & x > L/2. \end{array}\right. \end{eqnarray*}

For the DSM, there are propagating modes along the *y* and *z* directions. As shown in Fig. 2(b), a two-dimensional (2D) gapless phase for the DSM π junction with gapless dispersion along the *k_z_* direction is obtained numerically. (Actually, there is a tiny gap at the Dirac junction spectrum in the numerical calculation owing to the breaking of rotation symmetry.)

For the vortex configuration, we consider a flux along the (0, 0, 1) direction (*z* direction). The $\mathbf {\Delta (\mathbf {r})}$ can be written as


(5) \begin{eqnarray*} \mathbf {\Delta (\mathbf {r})}=\Delta (r)e^{i\theta }, \end{eqnarray*}

where *r* is the distance to the vortex line, θ is the polar angle and Δ(*r*) takes the form Δ(*r*) = Δ_0_Θ(*r* − *R*) with step function Θ(*r*) and core size *R*. Throughout the paper, we take Δ_0_ = 0.1 and use an infinitesimal value of *R*, and the qualitative results do not depend on the pairing function, the vortex profile and *R*, as shown in the online supplementary material. If we pinch all the π-junction slices into a vortex, the VBSs are dispersive along *k_z_*. As plotted in Fig. 2(c), there is a gapless VBS dispersion. This gapless feature has been obtained in DSMs [26–28] and WSMs [28,29].

**Figure 3.**
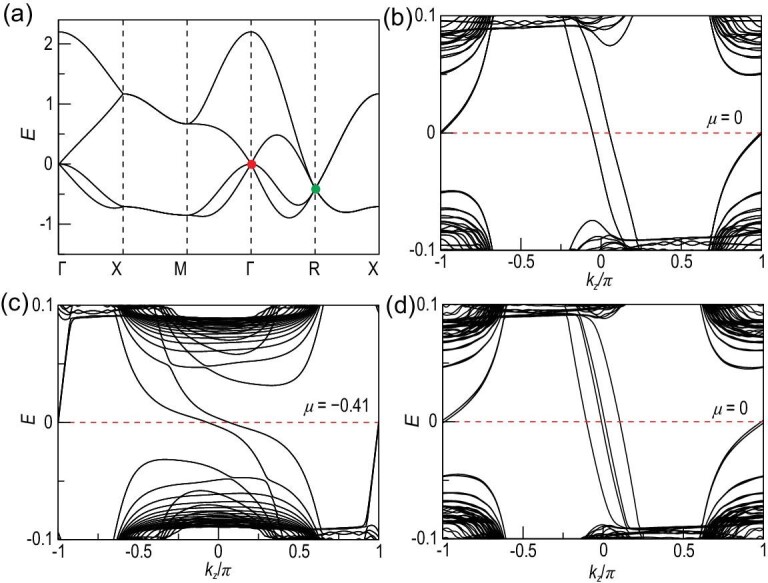
(a) Band structure for the chiral crystal eight-band model without SOC. There are one spin-1 excitation (red dot) around the Γ point and double-Weyl fermions (green dot) around the *R* point. (b) and (c) The vortex energy spectrum for (a) bands with μ = 0.0, −0.41, which are measured from the gapless point at the Γ point. Two double-degenerated chiral gapless vortex bound bands locate around *k_z_* = 0, while another branch of chiral bands locates around *k_z_* = π. (d) The vortex spectrum for the chiral crystal in the presence of SOC with μ at the RSW degenerate point. The gapless chiral bands are robust, while the double-degenerated chiral bands in (b) are split due to SOC.

There is also another large class of TSMs with higher-order massless fermions, named chiral crystals, like RhSi, CoSi, RhGe, CoGe, etc. [2,18–20,45]. For the chiral crystal, there is one long Fermi arc connecting the degeneracy points with Chern numbers C = 2, C = −2, which has been experimentally confirmed in CoSi [21,22]. In order to study the physics of chiral crystals, we apply a minimal eight-band model, which is the prototype model for RhSi (details can be found in the online supplementary material and [19]). The band structure for this model is plotted in Fig. 3(a). From the band structure, we can see that there are one C = 2 spin-1 excitation (red dot) around the Γ point and one C = −2 double-Weyl fermion around the *R* point (green dot) in the absence of spin-orbit coupling (SOC), which is a general feature for the chiral crystal. To study the vortex property, a magnetic flux along the (0,0,1) direction is inserted. The Hamiltonian for this case can be obtained by changing the *H_D_*(*k*) to *H_W_*(*k*), as defined in the online supplementary material, including all the parameters.

As discussed above, it is interesting to see how the VBSs behave around these special gapless points. We first put the chemical potential μ exactly at the spin-1 excitation point. In this case, the above condition for the specular reflection is satisfied. Hence, there should be gapless bound states inside the vortex, which is plotted in Fig. 3(b). There are two chiral vortex bands connecting the upper and lower bounds of the SC gap around *k_z_* = 0, as predicted. There is also another branch of chiral gapless bands around *k_z_* = π, which is related to the *R*-point degeneracy, as discussed later. If μ is moved to the double-Weyl points, similar gapless bound states are also obtained, as shown in Fig. 3(c). Interestingly, when μ sits between the spin-1 and double-Weyl degeneracy points, we find that gapless VBSs always exist. Furthermore, if μ is in the range −0.75 ≤ μ ≤ 0.59, we can always find VBSs with gapless chiral modes that disperse along the *z* direction. Moving away from this region, a gapped vortex is obtained.

We can extend the above calculation to the spin-3/2 RSW fermions. The spin-3/2 RSW fermion [7–9] can be obtained by introducing SOC into the chiral crystal tight-binding model [19]. The six-fold degeneracy at the Γ point is split into a spin-3/2 RSW point and a two-fold degenerate Weyl point. Upon introducing the vortex in the *z* direction, we again obtain the gapless chiral VBSs dispersing along the *z* direction when μ is at the RSW point, as shown in Fig. 3(d). Owing to SOC, the two spin-degenerated vortex bound bands split into four bound bands around the Γ point. The gapless chiral VBSs also exist as long as μ lies between μ_*c*1_ = −0.87 and μ_*c*2_ = 0.59.

## TOPOLOGICALLY PROTECTED CHIRAL VORTEX MODES FROM PUMPING

**Figure 4.**
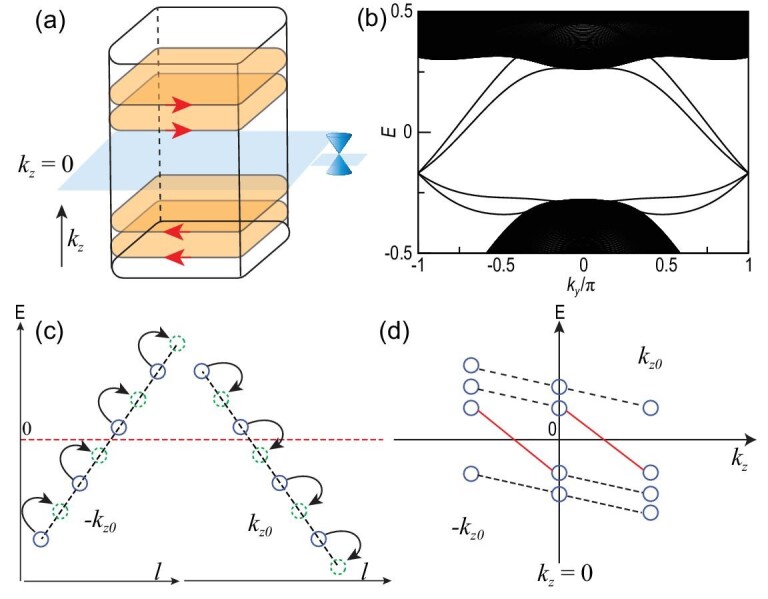
(a) Chiral edge modes above the *k_z_* = 0 plane and the opposite edge modes below the *k_z_* = 0 plane for the chiral crystal. (b) Chiral edge modes for the chiral crystal eight-band model in the presence of SOC with open boundary in the *x* direction for a constant *k_z_* plane with *k_z_* = 0.4π, where the mode propagates along *y* with the chemical potential set at the four-fold degenerate RSW point at Γ. (c) The chiral state energies (blue circles) in the ±*k*_*z*0_ planes and their shifts (green dashed circles) under topological pumping after flux insertion. Note that the chiral states at *k*_*z*0_ cross 0 under pumping. (d) Spectral flow for the vortex states. The spectra for ±*k_z_* are related to each other due to particle-hole symmetry. The bands connecting the ±*k*_*z*0_ with the *k_z_* = 0 plane give rise to the gapless chiral vortex bands (red lines). The connections between other states are plotted with black dashed lines.

All the above chiral VBSs are closely connected to the topological non-trivial band structures of the chiral crystal. The symmetry-protected RSW fermions around the Γ point and the Weyl fermions around the *R* point result in a long Fermi arc extending from the center to the corner of the surface Brillouin zone. The long Fermi arc also reflects the fact that, within each *k_z_* plane (0 < *k_z_* < π), the band structures of the chiral crystal describe a 2D quantum Hall insulator with Chern number C = 2, and for each −*k_z_* plane, the bands carry Chern number C = −2. The two Hamiltonians at *k_z_* and −*k_z_* are connected by time-reversal symmetry. Accordingly, there exist two chiral edge modes on the edge of each *k_z_* plane, and the chiral edge modes carry opposite chirality for the −*k_z_* planes, as indicated in Fig. 4(a) and calculated in Fig. 4(b).

We can use topological flux pumping to show that there is a one-to-one correspondence between these chiral edge modes and the chiral VBSs in the weak pairing limit [42,46]. For simplicity, we focus on one single chiral edge mode first and generalize to multiple chiral modes in the end. We consider the VBSs problem in the cylinder geometry with the vortex line parallel to the cylinder along the *z* direction. Under this condition, in the absence of the vortex line, the chiral edge modes can be described by a series of states *H*_chiral_(*k_z_*) = *v*(*k_z_*)*l* − μ with $l = \pm \frac{1}{2}, \pm \frac{3}{2},\ldots$ the angular momentum owing to rotation symmetry and the sign of *v*(*k_z_*) characterizing the chirality. Then, we can switch on a π flux going through the vortex line. The chiral edge modes become $H_{\text{chiral}}^{\text{vortex}}(k_z) = v(k_z) l^\prime -\mu$, with angular momentum increasing by one-half as $l^\prime = l + \frac{1}{2}$. Namely, each chiral edge mode is shifted by half of the minimal gap and the direction of the shift is determined by the chirality. Furthermore, the chiral edge modes on the edge of the *k_z_* plane and −*k_z_* plane carry opposite chirality due to time-reversal symmetry. Therefore, *H*_chiral_(*k_z_*) and *H*_chiral_(−*k_z_*) are both shifted half of the minimal gap but in opposite directions after the π flux is inserted.

For a chiral crystal, we can always find one insulating *k*_*z*0_ plane. Furthermore, each chiral edge mode in the *k*_*z*0_ plane combined with its time-reversal partner in the −*k*_*z*0_ plane contributes to a single state that goes across the Fermi energy after the π flux is inserted, as illustrated in Fig. 4(c). This pumping process can be described by the index *N*(*k*_*z*0_) − *N*(−*k*_*z*0_) = −*sgn*(*v*(*k*_*z*0_)), where *N*(*k_z_*) is the number of states below the Fermi energy at *k_z_*. For the superconducting state, an infinitesimal pairing cannot change the above process, and the π flux not only pumps an electron but also pump a hole; see the online supplementary material for further details. Therefore, in the superconducting state the above index is doubled, namely *N*_chiral_ = *N*_sc_(*k*_*z*0_) − *N*_sc_(−*k*_*z*0_) = −2*sgn*(*v*(*k*_*z*0_)), where *N*_sc_(*k_z_*) is the number of the vortex bound states with negative energy at *k_z_*. Now, we can consider the spectral flow of the vortex bound states. Owing to the particle-hole symmetry, the energy spectrum of bound states for *k_z_* is opposite to −*k_z_*. Then, the energy spectrum for *k_z_* = 0 is exactly particle-hole symmetric. For instance, we take four states as one example, which leads to two positive states and two negative energy states at *k_z_* = 0, as shown in Fig. 4(d). For *k*_*z*0_, there are one positive state and three negative energy states owing to finite *N*_chiral_. Hence, there are two chiral vortex modes connecting the *k_z_* = 0 plane to the ±*k*_*z*0_ plane, as illustrated in Fig. 4(d). Obviously, *N*_chiral_ is just the number of chiral vortex modes between *k*_*z*0_ and −*k*_*z*0_. Considering the vorticity of the vortex and the number of chiral edge modes, the above topological index can be generalized to *N*_chiral_ = −2ηC(*k_z_*), with η the vorticity of the vortex and C(*k_z_*) the Chern number relating to the number of chiral modes in the *k_z_* plane.

Note that in the above analysis we assume an insulating gap in the *k*_*z*0_ plane in the normal state. For the chiral crystal, for any chemical potential μ in between $E_\Gamma$ and *E_R_*, with $E_\Gamma$ and *E_R_* the energies of the degenerate points at Γ and *R*, respectively, we can always find an insulating gap in some *k*_*z*0_ plane. Therefore, for any chemical potential satisfying $E_R < \mu < E_\Gamma$, there are always four chiral vortex modes near *k_z_* = 0 and four near *k_z_* = π. The topological pumping process can be directly generalized to Weyl semimetals. As illustrated in Fig. 2(a), the most important property for Weyl semimetals are the Weyl points containing the spin-1/2 Weyl fermions with monopole charge C = 1. Similar to the chiral crystals, we can always find one C ≠ 0 insulating the *k* plane with chiral edge modes, where this plane sits between two Weyl points with opposite chirality. Based on the above analysis, the π-flux pumping gives rise to chiral gapless vortex bound states in the superconducting Weyl semimetal [28,29].

It is worth mentioning that the chiral gapless vortex modes in the superconducting chiral crystals and Weyl semimetals are protected by the translational symmetry along the vortex line, which turns out to be rather robust. This stems from the fact that the chiral modes can be gapped out only when two modes with opposite chirality hybridize, i.e. the translational symmetry is broken, which may occur in the presence of disorder. This is very different from the Dirac semimetal case where the rotational symmetry is also vital for the helical gapless vortex modes [26,27]. The difference is in accordance with normal band topology. The Weyl point can be gapped out only when two Weyl points with opposite monopole charges hybridize, while rotational symmetry is necessary to stabilize the charge-neutral Dirac points.

Additionally, we also studied VBSs in anisotropic Weyl semimetals, tilted and type-II Weyl semimetals [47], etc. All of these semimetals contain gapless VBSs, as we expected. More details can be found in the online supplementary material. We also want to mention that the gapless vortex bound states can also be found in other cases. For example, in the doped superconducting topological insulator, the vortex states can undergo a topological phase transition, which will become gapless at the phase transition point [48–50]. In fact, at the critical point, the physics is equivalent to that in Weyl semimetals. Experimentally, whether the SC system with vortex is gapless or gapped can be detected by optical responses such as the optical conductivity σ(ω) or thermodynamic experiments such as heat capacity. For example, if the vortex is gapped, σ(ω) shows an absorption edge at the mini-gap of the Caroli-de Gennes-Matricon states, while a continuum spectrum is obtained if the vortex is gapless.

## CONCLUSION

In summary, we study the gapless vortex bound states for the superconducting topological semimetals with non-trivial fermionic excitations. Owing to their fermionic excitations, the topological semimetals always host the specular Andreev reflection, which gives rise to the propagating Andreev modes inside the TSM π-phase SNS junctions. Furthermore, the vortex can be mapped to the superposition of π-SNS junctions. Therefore, a gapless vortex bound state can be obtained when the chemical potential sits at the degenerate points. Beyond the previously studied Dirac and Weyl semimetal gapless vortex solutions, we generalize the above discussion to chiral crystals that hold exotic gapless fermions, such as the quasi-spin-1 Weyl fermion and the spin-3/2 Rarita-Schwinger-Weyl fermion. The gapless chiral vortex bound states exist in a large region of the chemical potential. These chiral modes are topologically protected and closely related to the topologically non-trivial band structure of the chiral crystal, which can be fully understood from the topological pumping process. Our theory paves a new way to the understanding of vortex bound states in all the superconducting topological semimetals. It is also hoped that our findings will further stimulate the investigation for superconducting topological semimetals, such as RhGe, which is a highly possible example for a superconducting chiral crystal [45].

## Supplementary Material

nwac121_Supplemental_File
