# Supplementary material for: Gapless vortex bound states in superconducting topological semimetals
Source: Natl Sci Rev. 2022 Jun 24;10(2):nwac121. doi: 10.1093/nsr/nwac121 (PMC10016197; doi:10.1093/nsr/nwac121)
Supplement: nwac121_Supplemental_File [file nwac121_supplemental_file.pdf]

Supplemental material for Gapless vortex bound states in superconducting topological semimetals

Yi Zhang,[1,2,*] Shengshan Qin,[2,*] Kun Jiang,[3,†] and Jiangping Hu[3,4,2,‡]

[1] *Department of Physics, Shanghai University, Shanghai 200444, China*
[2] *Kavli Institute of Theoretical Sciences, University of Chinese Academy of Sciences, Beijing, 100190, China*
[3] *Beijing National Laboratory for Condensed Matter Physics and Institute of Physics, Chinese Academy of Sciences, Beijing 100190, China*
[4] *Collaborative Innovation Center of Quantum Matter, Beijing 100190, China*

(Dated: May 29, 2022)

A. Model Hamiltonian for chiral crystal

To describe the band structure of the chiral crystal, we adopt the tight-binding model for RhSi introduced in Ref. 1. This model considers a simple cubic lattice with four sites per unit cell (A,B,C,D), with Cartesian coordinates $\mathbf{r}_A$=(0,0,0), $\mathbf{r}_B=(\frac{1}{2},\frac{1}{2},0)$, $\mathbf{r}_C=(\frac{1}{2},0,\frac{1}{2})$ and $\mathbf{r}_D=(0,\frac{1}{2},\frac{1}{2})$ and each site carries a spin-1/2 orbital, thus describing an eight-band model. The total Hamiltonian can be written as

$$H_W(\mathbf{k}) = H_0(\mathbf{k}) + V_2(\mathbf{k}) + \sum_{i=1,3} (V_{r,i}(\mathbf{k}) + V_{s,i}(\mathbf{k})) \tag{S1}$$

where

$$\begin{aligned} H_0(\mathbf{k}) = v_1 &\left[\tau^x \cos\left(\frac{k_x}{2}\right)\cos\left(\frac{k_y}{2}\right) + \tau^x\mu^x \cos\left(\frac{k_y}{2}\right)\cos\left(\frac{k_z}{2}\right) + \mu^x \cos\left(\frac{k_z}{2}\right)\cos\left(\frac{k_x}{2}\right)\right] \\ + v_p &\left[\tau^y\mu^z \cos\left(\frac{k_x}{2}\right)\sin\left(\frac{k_y}{2}\right) + \tau^y\mu^x \cos\left(\frac{k_y}{2}\right)\sin\left(\frac{k_z}{2}\right) + \mu^y \cos\left(\frac{k_z}{2}\right)\sin\left(\frac{k_x}{2}\right)\right] \end{aligned} \tag{S2}$$

$$V_2(\mathbf{k}) = v_2\left[\cos(k_x) + \cos(k_y) + \cos(k_z)\right] \tag{S3}$$

are the spinless parts and

$$V_{r1}(\mathbf{k}) = v_{r1}\left[\tau^y\mu^z\sigma^y \cos\left(\frac{k_x}{2}\right)\cos\left(\frac{k_y}{2}\right) + \tau^y\mu^x\sigma^z \cos\left(\frac{k_y}{2}\right)\cos\left(\frac{k_z}{2}\right) + \mu^y\sigma^x \cos\left(\frac{k_z}{2}\right)\cos\left(\frac{k_x}{2}\right)\right] \tag{S4}$$

$$V_{r2}(\mathbf{k}) = v_{r2}\left[\tau^y\sigma^z \cos\left(\frac{k_x}{2}\right)\cos\left(\frac{k_y}{2}\right) + \tau^x\mu^y\sigma^x \cos\left(\frac{k_y}{2}\right)\cos\left(\frac{k_z}{2}\right) + \tau^z\mu^y\sigma^y \cos\left(\frac{k_z}{2}\right)\cos\left(\frac{k_x}{2}\right)\right] \tag{S5}$$

$$V_{r3}(\mathbf{k}) = v_{r3}\left[\tau^y\mu^z\sigma^x \sin\left(\frac{k_x}{2}\right)\sin\left(\frac{k_y}{2}\right) + \tau^y\mu^x\sigma^y \sin\left(\frac{k_y}{2}\right)\sin\left(\frac{k_z}{2}\right) + \mu^y\sigma^z \sin\left(\frac{k_z}{2}\right)\sin\left(\frac{k_x}{2}\right)\right] \tag{S6}$$

$$V_{s1}(\mathbf{k}) = v_{s1}\left[\tau^x\sigma^x \sin\left(\frac{k_x}{2}\right)\cos\left(\frac{k_y}{2}\right) + \tau^x\mu^x\sigma^y \sin\left(\frac{k_y}{2}\right)\cos\left(\frac{k_z}{2}\right) + \mu^x\sigma^z \sin\left(\frac{k_z}{2}\right)\cos\left(\frac{k_x}{2}\right)\right] \tag{S7}$$

$$V_{s2}(\mathbf{k}) = v_{s2}\left[\tau^x\sigma^y \cos\left(\frac{k_x}{2}\right)\sin\left(\frac{k_y}{2}\right) + \tau^x\mu^x\sigma^z \cos\left(\frac{k_y}{2}\right)\sin\left(\frac{k_z}{2}\right) + \mu^x\sigma^x \cos\left(\frac{k_z}{2}\right)\sin\left(\frac{k_x}{2}\right)\right] \tag{S8}$$

$$V_{s3}(\mathbf{k}) = v_{s3}\left[\tau^x\mu^z\sigma^z \cos\left(\frac{k_x}{2}\right)\sin\left(\frac{k_y}{2}\right) - \tau^y\mu^y\sigma^x \cos\left(\frac{k_y}{2}\right)\sin\left(\frac{k_z}{2}\right) + \tau^z\mu^x\sigma^y \cos\left(\frac{k_z}{2}\right)\sin\left(\frac{k_x}{2}\right)\right] \tag{S9}$$

describe the spin orbit coupling terms. Here τ, μ and σ are Pauli matrices with τ matrices describing the hopping between the A and B and the C and D sites, μ matrices describing the hopping between the A and C and the B and D sites and σ matrices describing the onsite spin-1/2 orbital. In our calculation, we take the parameters (in units of eV) v_1=0.55, v_p=-0.76, v_2=0.16 for the case without SOC, while for the case with SOC, we use v_{r2}=-0.1 to describe the additional SOC term. The band structure in the presence of SOC is shown in Fig. S1

---

*These two authors contributed equally
†Electronic address: jiangkun@iphy.ac.cn
‡Electronic address: jphu@iphy.ac.cn

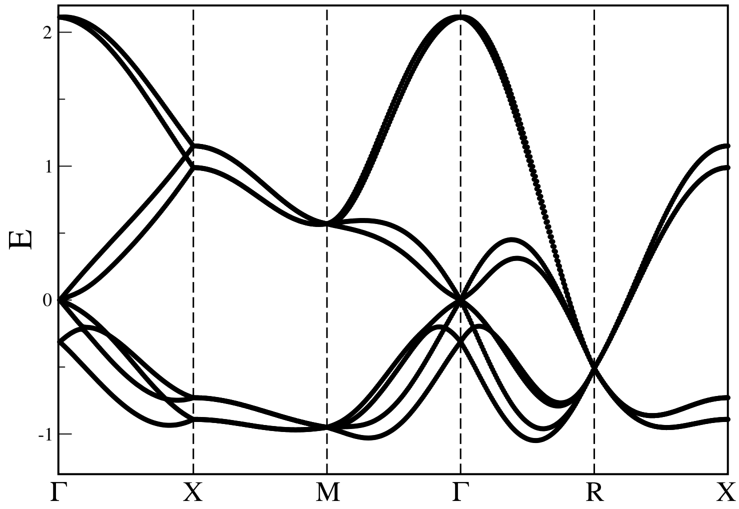

FIG. S1: Band structure for the chiral crystal eight-band model with SOC described by v_{r2}=-0.1. The spin-1 excitation at Γ splits into a four-fold degenerate spin-3/2 Rarita-Schwinger-Weyl (RSW) point and a double degenerate point.

B. Vortex bound states for different chemical potential

In this section, we show the vortex bound states with and without SOC for different chemical potential in Fig. S2 and Fig. S3, which shows the emergence and disappearance of the chiral gapless vortex modes singling the topological phase transitions as tuning the chemical potential. The μ_{c1} and μ_{c2} label the critical values for the gap to gapless transition. The phase diagrams as a function of chemical potential μ involving the states with and without gapless vortex bound states are also shown in the figures.

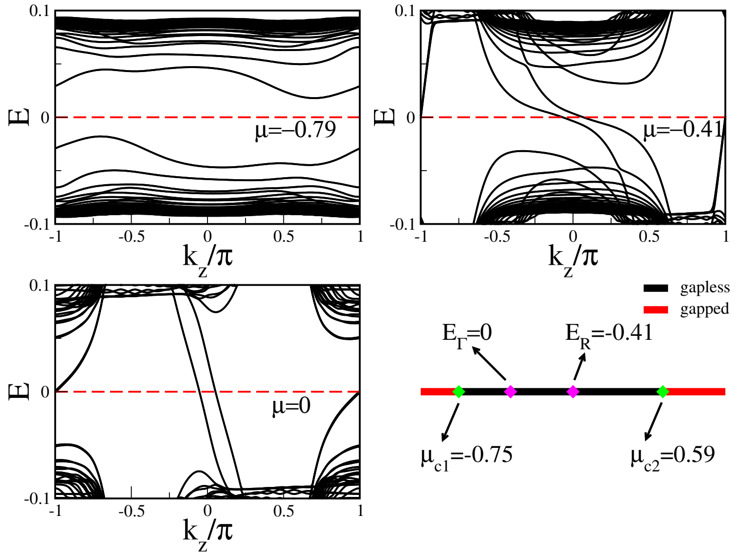

FIG. S2: The vortex spectrum for the chiral crystal without SOC for three different chemical potential with μ=-0.79, -0.41 and 0 measured from the 2×3 fold degenerate gapless point as well as the phase diagram of the vortex bound states as a function the chemical potential μ, showing the two critical values μ_{c1} and μ_{c2}. The energy of the two special bulk gapless points are also shown as E_Γ and E_R for comparison.

C. Topological pump in the superconducting state

In the main text, we claim that there exit N_{chiral} chiral vortex modes in between k_z and $-k_z$ with $N_{\text{chiral}} = -2\eta C(k_z)$. It stems from that the π flux not only pumps an electron but also pumps a hole. This can be directly

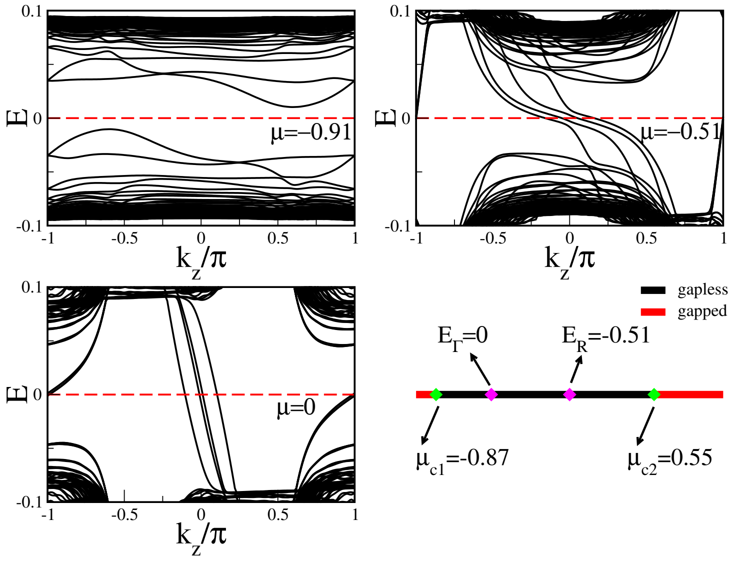

FIG. S3: The vortex spectrum for the chiral crystal in the presence of SOC for three different chemical potential with μ=-0.91, -0.51 and 0 measured from the 2×3 fold degenerate gapless point as well as the phase diagram of the vortex bound states as a function the chemical potential μ, showing the two critical values μ_{c1} and μ_{c2}. The energy of the two special bulk gapless points are also shown as E_Γ and E_R for comparison.

seen by considering the particle-hole symmetry in the superconducting state. Owing to the particle-hole symmetry under BdG equations, one state with angular momentum l at k_z is mapped to another state with angular momentum $-l$ at $-k_z$, as illustrated in Fig.S4. By inserting a π flux, if an electron is pumped to the negative energy at k_z as shown in the Fig.S4 upper panel, the particle-hole symmetry ensures that one hole must be pumped to the positive energy at $-k_z$ as shown in the Fig.S4 down panel. Namely, the number of the states with negative energy satisfies $N_{\rm sc}(k_z) - N_{\rm sc}(-k_z) = -2sgn(v(k_z))$ and $N_{\rm chiral} = -2\eta C(k_z)$ chiral vortex modes arise in between k_z and $-k_z$.

D. Vortex bound states with different vorticities

As shown in the main text, the gapless chiral vortex bound states can be understood from the topological pumping process by inserting a flux into the system which leads to the number of the gapless chiral mode $N_{\rm chiral} = -2\eta C(k_z)$, with η the vorticity of the vortex and $C(k_z)$ the Chern number relating to the number of chiral modes in the k_z plane. This means that the number of the gapless chiral modes is proportional to the vorticity. Indeed, we see that the number of gapless modes increases from 4 to 8 as the vorticity increases from 1 to 2, compared with the results in the main text as shown in Fig. S5 , which further validates the understanding from the topological pumping.

E. Vortex bound states dependence on vortex core sizes and pairing profiles

In the main text, we use the infinitesimal value of core size R which is equivalent to R=0.5 since the shortest distance between two sites projecting to the xy plane is 0.5. Here the gapless vortex bound states for various values of R are also presented in Fig. S6**a**, which show the gapless dispersion is stable for larger values of R. Moreover, we also study the case for pairing profile function in the hyper tangent form $\Delta(r) = \Delta_0 \tanh(r/R)$ instead of the form $\Delta(r) = \Delta_0 \Theta(r - R)$ used in the main text, which shows similar gapless vortex bound states shown in Fig. S6**b**. Therefore, the gapless modes inside the topological semimetal vortex don't qualitatively depend on the vortex core size R and pairing proflie $\Delta(r)$.

F. Localization of the vortex bound states

To confirm whether the gapless bound states are bounded around the vortex, we plot the wavefunction norm square $|\psi(x, y)|^2$ in the xy plane in Fig. S7 for the zero energy mode shown in Fig. S2 with μ=0. From Fig. S7, we found

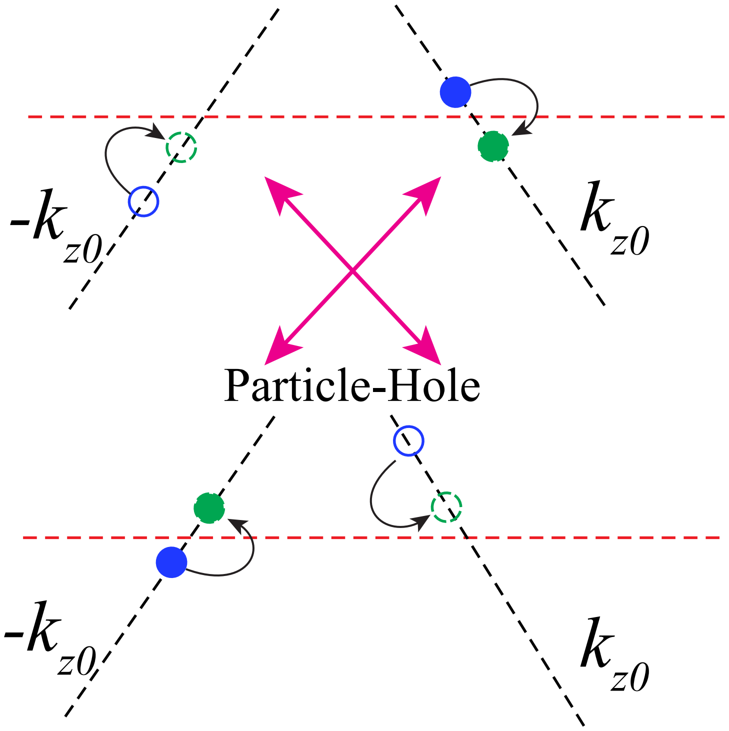

FIG. S4: The pumping process and its particle-hole related partner under BdG. The (l, k_z) state with energy E is mapped to another state with energy $-E$ at $(-l, -k_z)$ under particle-hole transformation. For example, the solid blue state at k_{z0} in the upper panel is mapped to another solid blue state at $-k_{z0}$ in the lower panel . After inserting one flux into the system, the blue circles are pumped to the green circles. Therefore, if (l_0, k_{z0}) state is pumped across the Fermi level, there must be another hole state is also pumped across the Fermi level at $(-l_0, -k_{z0})$.

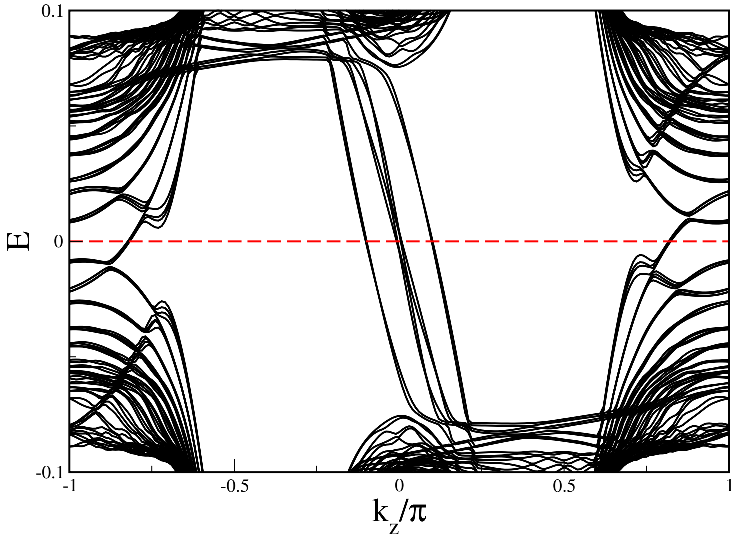

FIG. S5: The vortex spectrum for the chiral crystal in the presence of SOC with μ at the RSW degenerate point for a vortex with vorticity η=2. The number of gapless modes increases from 4 to 8 compared with the case with vorticity η=1 in the main text.

that the zero energy mode is localized around the vortex core with localization length around 1.5. Since the system has translation invariance along z direction, it is also extended along the z direction.

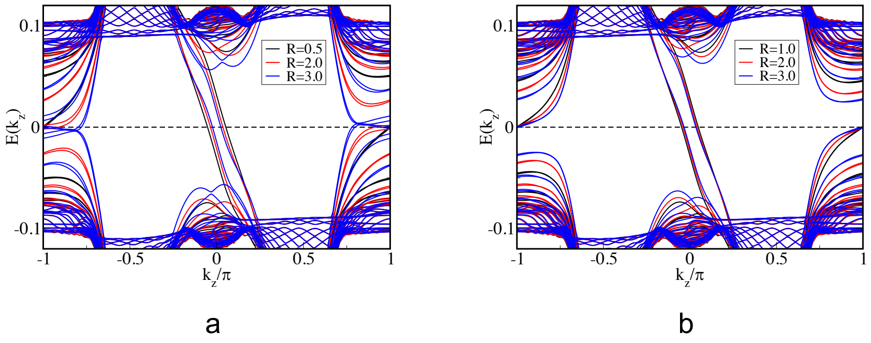

FIG. S6: **a**, The energy dispersions of the vortex bound states for the chiral crystal model along the z direction for μ=0.0 with three different vortex core size R=0.5, 2.0, 3.0. **b**, The energy dispersions for the vortex function $\Delta(r) = \Delta_0 \tanh(r/R)$ with three different values of R=1.0,2.0,3.0.

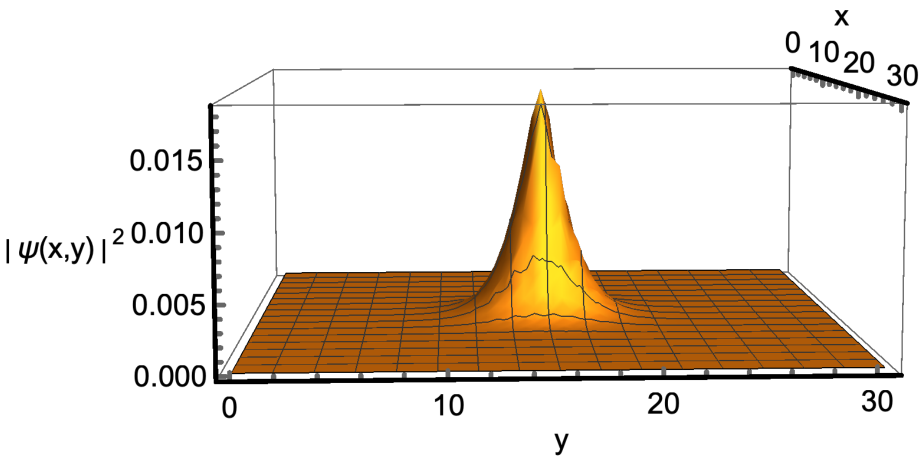

FIG. S7: The distribution of the wavefunction square $|\psi(x,y)|^2$ for the zero-energy mode in Fig. S2 with μ=0. This mode is localized around the vortex center at (15,15).

G. Vortex bound states for various types of Weyl semimetals

In this section, we studied the vortex bound states for various types of Weyl semimetals, to demonstrate the universality of the gapless property. We first introduce the lattice model to realize these Weyl dispersions which has the following form:

$$\begin{aligned} H(k_x,k_y,k_z) &= (m - t_1 cosk_x - t_2 cosk_y - t_3 cosk_z)\sigma_z s_0 + t_1' \sin k_x \sigma_x s_x + t_2' \sin k_y \sigma_x s_y \\ &+ t_3' \sin k_z (\cos k_x + \cos k_y - 2)\sigma_y s_0 + a_1 \sin k_z \sigma_0 s_z + a_2 \cos k_z \sigma_0 s_0 \end{aligned} \tag{S10}$$

where the Pauli matrices are defined as same as the main text. Here, we choose the parameters as $(m, t_1, t_2, t_3, t_1', t_2', t_3', a_1, a_2)$=(2.2,1.0,1.0,0.5,1.0,1.0,1.0,0,0). Without the last two terms with coefficients a_1 and a_2, the model describes two accidental Dirac points at k=(0,0,$\pm$arccos(0.4)). Below, the study the vortex bound state with pairing amplitude Δ_0=0.2 in form of $\Delta(r) = \Delta_0 \Theta(r-R)$ with infinitesimal core size R.

1. *Type-I Weyl semimetal*

Introducing a finite a_1 term with a_1=0.2 splits each Dirac point into two Weyl points at (0,0,$\pm k_{c1}$) and (0,0,$\pm k_{c2}$) with k_{c1}=arccos(20/29) and k_{c2}=$\pi/2$. The band dispersion along the z direction with k=(0,0,k_z) is shown in Fig. S8**a**. For each k_z, there are four bands along the (k_x,k_y) plane. The Chern number can be defined for each of these four

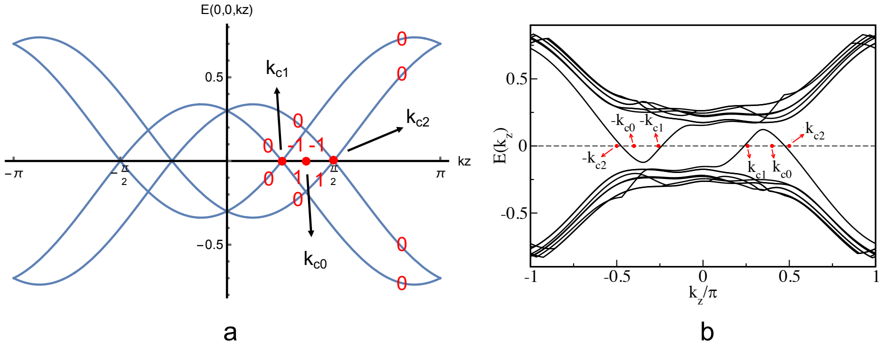

FIG. S8: **a**, The band dispersion for the constructed Weyl semimetal in Eq. S10 along the z direction together with the Chern number evolution (red numbers) before and after crossing the two Weyl points (0,0,k_{c1}) and (0,0,k_{c2}). **b**, The dispersion of the vortex bound states along the z direction for μ=0.0 showing the gapless dispersion for the constructed Weyl semimetal, where the locations of the Weyl points in the normal bands as well as the special momentum k_{c0} are labeled by the red dots.

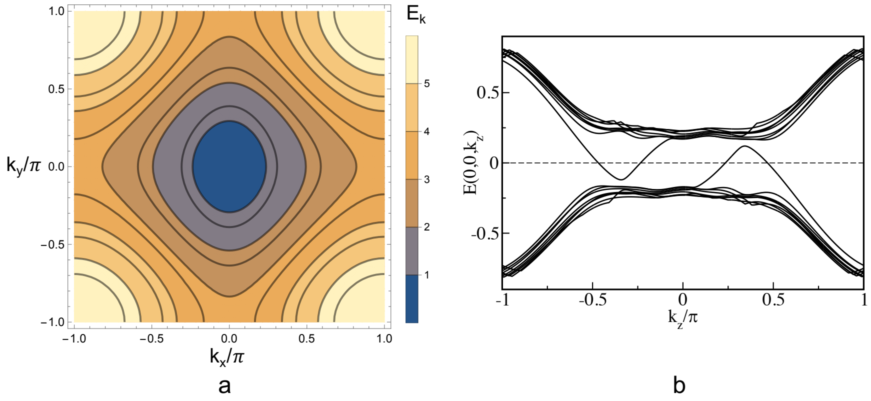

FIG. S9: **a**, Equal-energy contours of the anisotropic Weyl semimetal for various energy at the $k_z = k_{c2}$ plane, which breaks the C_{4z} symmetry. **b**, The dispersion of the vortex bound states along the z direction for μ=0.0 showing the gapless dispersion for the anisotropic Weyl semimetal.

bands. In Fig. S8**a**, we also plot the Chern number evolution for bands before and after the band crossing at the Weyl points. We can always find a $k_z = k_{c0}$ between $k_z = k_{c1}$ and $k_z = k_{c2}$ where the bulk dispersion at this k_z plane is fully gapped with an edge mode due to the finite Chern number. Then using the same flux pumping argument, we can conclude that if the chemical potential μ inside the insulating gap, the vortex bound states have to cross zero energy twice with one between k_z=0 and k_z=k_{c0} and the other between $k_z = k_{c0}$ and $k_z = \pi$. To verify this point, we calculated the band dispersions with chemical potential μ=0.0 as shown in Fig. S8**b**.

2. Anisotropic Weyl semimetal

We can also study the anisotropic Weyl semimetal. We set t'_2=0.5 to break the C_{4z} symmetry which makes the two Weyl points have anisotropic dispersion in the xy plane. Fig. S9**a** shows the equal-energy contours of the modified model at the k_{c2} plane for various Fermi energy which clearly shows the C_{4z} breaking feature. As shown in Fig. S9**b**, we find that the gapless vortex bound states are robust against the anisotropy introduced here.

3. Tilted Weyl semimetal

To study the case with tilted Weyl semimetal, we set t'_2 back to 1.0 and further introduce the extra term $a_2 \cos k_z \sigma_0 s_0$ which tilts the dispersion along the z direction. For a_2=0.3, the band dispersion along the z direction is significantly tilted as shown in Fig. S10**a**,**b** but not strong enough to form a type-II Weyl semimetal. Using the same flux pumping

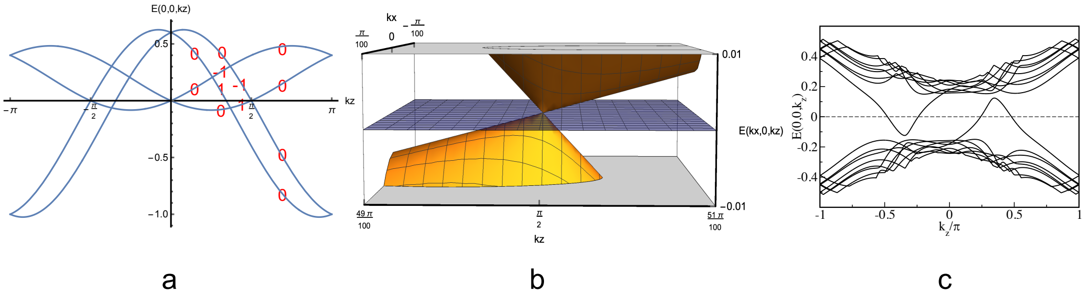

FIG. S10: **a**, The band dispersion for the tilted Weyl semi-metal along the z direction together with the Chern number evolution (red numbers) before and after crossing the two Weyl points k_{c1} and k_{c2}. **b**, The bulk band dispersion around the Weyl point (0,0,$\pi/2$) in the xz plane, where the dispersion along the k_z direction is tilted. The zero-energy plane is also plotted for clarification. **c**, The dispersion of the vortex bound states along the z direction for μ=0.2 showing the gapless dispersion for the tilted Weyl semimetal.

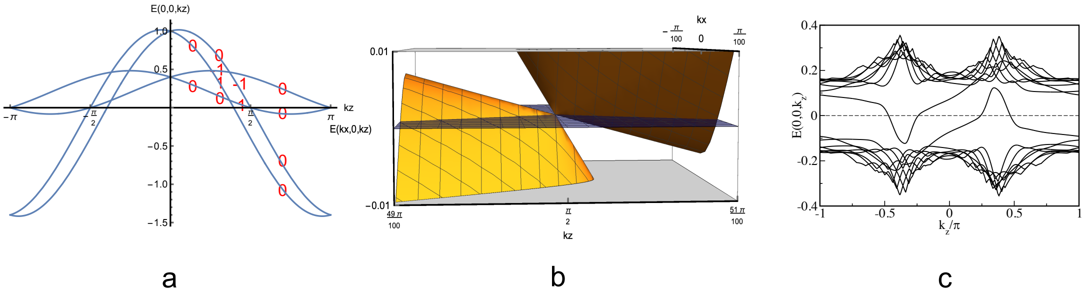

FIG. S11: **a**, The band dispersion for the type-II Weyl semimetal along the z direction together with the Chern number evolution (red numbers) before and after crossing the two Weyl points k_{c1} and k_{c2}. **b**, The bulk band dispersion around the Weyl point (0,0,$\pi/2$) in the xz plane, where the dispersion along the k_z direction is tilted. The zero-energy plane is also plotted for clarification. **c**, The dispersion of the vortex bound states along the z direction for μ=0.3 showing the gapless dispersion for the type-II Weyl semimetal.

argument as above, we also find the existence of the gapless vortex bound states in this case of tilted Weyl semimetal, which is shown numerically in Fig. S10**c** with chemical potential μ=0.2.

4. Type-II Weyl semimetal

Moreover, to study the case with type-II Weyl semimetal, we further increase a_2=0.7, which tilts the band enough to realize a type-II Weyl semimetal. The band dispersion along the z direction and around the Weyl point in the xz plane are shown in Fig. S11**a**,**b**. The same flux pumping argument still applies in this case, and we can demonstrate the existence of the gapless vortex bound states in this case of type-II Weyl semimetal, which is shown numerically in Fig. S11**c** chemical potential μ=0.3.

5. Vortex with extended s-wave pairing function

In the main text, we foucs on the on-site s-wave case. The actual pairing function is irrelavent to the conclusion of the gapless vortex bound states as long as the superconducting spectrum is fully gapped. In order to demonstrate that, we calculate the vortex bound states for the constructed Weyl semimetal model in Eq. S10 with a_1=0.2 with extended s-wave vortex pairing function $\Delta(k) = \Delta_{s\pm}(\cos k_x + \cos k_y)$ in the xy plane with amplitude $\Delta_{s\pm}$=0.1. As shown in Fig. S12, the gapless bound states still exist in this case.

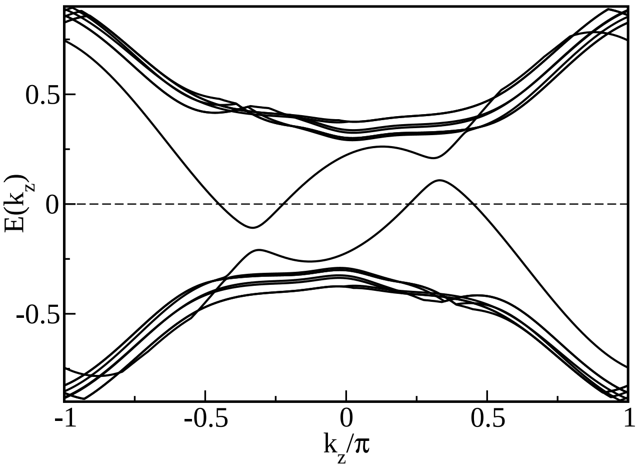

FIG. S12: The energy dispersion of the vortex bound states for the constructed Weyl semimetal in Eq. S10 along the z direction for μ=0.0 with extended s-wave pairing function $\Delta(k) = \Delta_{s\pm}(\cos k_x + \cos k_y)$.

[1] G. Chang et al., Phys. Rev. Lett. **119**, 206401 (2017).
